# Outcomes of Neoadjuvant Chemotherapy in Locally Advanced Oral Cancers: A Retrospective Analytical Study

**DOI:** 10.7759/cureus.71248

**Published:** 2024-10-11

**Authors:** Monesha Baskaran, S.M. Azeem Mohiyuddin, G. N. Manjunath, A. Sagayaraj, M. Kouser, Ravindra P Deo, Anil K Sakalecha, Kalyani Raju, Sampath Kumar M.N.

**Affiliations:** 1 Otolaryngology - Head and Neck Surgery, Sri Devaraj Urs Medical College, Kolar, IND; 2 Radiation Oncology, Sri Devaraj Urs Medical College, Kolar, IND; 3 Radio-Diagnosis, Sri Devaraj Urs Medical College, Kolar, IND; 4 Pathology, Sri Devraj Urs Medical College, Kolar, IND; 5 Oncology, Sri Devaraj Urs Medical College, Kolar, IND

**Keywords:** compartment clearance of infratemporal fossa, composite resection, loco-regional control, neck dissection, neoadjuvant chemotherapy, oral squamous cell carcinoma, recurrence

## Abstract

Introduction

Head and neck cancers account for 30% of malignancies in India and oral squamous cell carcinoma is most common. 80% of the patients present with locally advanced disease and many of them are inoperable. In this study we documented the outcomes of neoadjuvant chemotherapy (NACT) in locally advanced oral cancer staged T4 with regards to downstaging the disease and to make the tumor more amenable for surgery with better access.

The objectives of the study are to document the outcomes of NACT in patients with locally advanced oral cancers and to correlate the response to NACT and adequacy of resection by histopathology examination of the specimen.

Methodology

Clinical data of 96 patients with locally advanced squamous cell carcinoma of the oral cavity staged T4a and T4bwho received two cycles of NACT between December 2018 and November 2022 were reviewed respectively and the outcomes and response following NACT were documented.

Results

Among 96 patients in this study, 68 (70.9%) patients were staged T4a and 28 (29.1%) were staged T4b. 31 (30.2%) patients showed partial response, 36 (37.5%) had stable disease and 29 (30.2%) had progressive disease following 2 cycles of NACT. All patients underwent definitive surgery following NACT and were followed up for 14 months. Among those staged T4a, 12 patients (33.3%) had close margins, eight patients (34.8%) had positive margins and 10 patients (16.2%) had a recurrence. Among those staged T4b, 24 patients (66.7%) had close margins (less than 5mm distance after formalin fixation), 15 patients (65.2%) had positive margins while 17 patients (60.7%) had a recurrence in the 14 month follow-up period.

Conclusion

NACT makes tumors more amenable for surgery in locally advanced oral squamous carcinoma. However, 30% of patients do not respond to it.

## Introduction

Despite a downward trend in the overall prevalence of oral squamous cell carcinoma (OSCC) over the past few decades in most industrialized countries, it remains a common malignancy in both males and females in south-central Asia and central and eastern Europe. According to data from the Indian Council of Medical Research (ICMR), oral cancer ranks second in incidence of cancer among women (10.4%) and is the most common cancer among men (16.1%). Oral cancer is becoming more common and hence it is imperative to strengthen the diagnostic and therapeutic methods [1]. Buccal mucosa cancers and lower gingivobuccal sulcus complex cancers are more common among oral squamous cell carcinomas in our area owing to tobacco quid chewing and betel quid chewing [2]. The majority of patients are women from low socioeconomic backgrounds who have locally advanced diseases that are either inoperable or necessitate extensive and mutilating surgery, significant morbidity from adjuvant treatment, and poor quality of life. This female preponderance has been attributed to tobacco quid chewing and their propensity to hold the quid under their cheeks for extended periods, whereas the male population is more addicted to tobacco smoking. The mainstay of treatment for individuals with OSCC is the composite resection of the tumor (with skin and mandible in some cases), neck dissection, compartment clearance of infratemporal fossa (ITF) in T4b tumors, and complex reconstruction. The stage of the tumor, the state of the resected margin, the depth of invasion, and the presence of cervical nodal metastases are important prognostic markers that impact these individuals.

Adjuvant treatment may be in the form of radiotherapy with or without concurrent chemotherapy, with concurrent chemotherapy adding substantial side effects and morbidity to adjuvant treatment. However, in spite of such vigorous multimodality treatment, recurrences are still prevalent in such locally advanced tumors. Some of the surgical complications include flap necrosis, wound breakdown, oro-cutaneous fistula, infection, bleeding and blood vessel blowouts, chylous fistula, nerve damage, etc. The dreaded complications of chemotherapy include neurotoxicity, ototoxicity, nephrotoxicity, febrile neutropenia, and suppression of the bone marrow. Radiotherapy effects include wound breakdown, cutaneous desquamation, mucositis, and osteoradionecrosis. Only recently the overall and disease-free survival has marginally improved in this dreaded disease.

Neoadjuvant chemotherapy (NACT) refers to chemotherapy administered before surgery. Its role in the treatment of head and neck cancers is still controversial [3]. It reduces the distant recurrence rate from 38% to 14% in advanced stages of oral carcinomas [4]. The use of NACT for locally advanced oral malignancies has generated debate in the literature; some authors believe it improves the prognosis, while others do not [5-6].

The objectives of the study are to document the outcomes of NACT in patients with locally advanced oral squamous cell cancers and to correlate the response to NACT and adequacy of resection by histopathology examination of the specimen.

## Materials and methods

This was a retrospective analytical study approved by the Institutional Ethics Committee of Sri Devaraj Urs Medical College, Kolar, India with IEC no. SDUMC/KLR/IEC/519/2023-24.This study included a total of 96 patients with biopsy-proven squamous cell carcinoma of oral cavity staged T4a or T4b who received NACT followed by definitive surgery (composite resection + infratemporal compartment clearance in T4b tumors) + neck dissection + reconstruction. They also received postoperative adjuvant treatment in the form of radiotherapy or chemotherapy with radiotherapy. Consent was obtained from all the participants for being a part of the study.

Inclusion criteria

Patients between 40 to 70 years of age with biopsy proved squamous cell carcinoma of oral cavity staged T4a or T4b who received two cycles of NACT with cisplatin (100mg/m^2^) and paclitaxel (175mg/m^2^) once in 3 weeks and underwent definitive surgery in the form of composite resection with neck dissection and ITF clearance whenever ITF was involved and reconstruction and later completed adjuvant treatment in the form of radiotherapy/chemotherapy with radiotherapy and had a minimum follow-up of 6 months.

Exclusion criteria

Patients with second primary tumors, recurrent OSCC, or those who received chemotherapy or radiotherapy to the head and neck earlier were excluded from the study.

**Figure 1:**
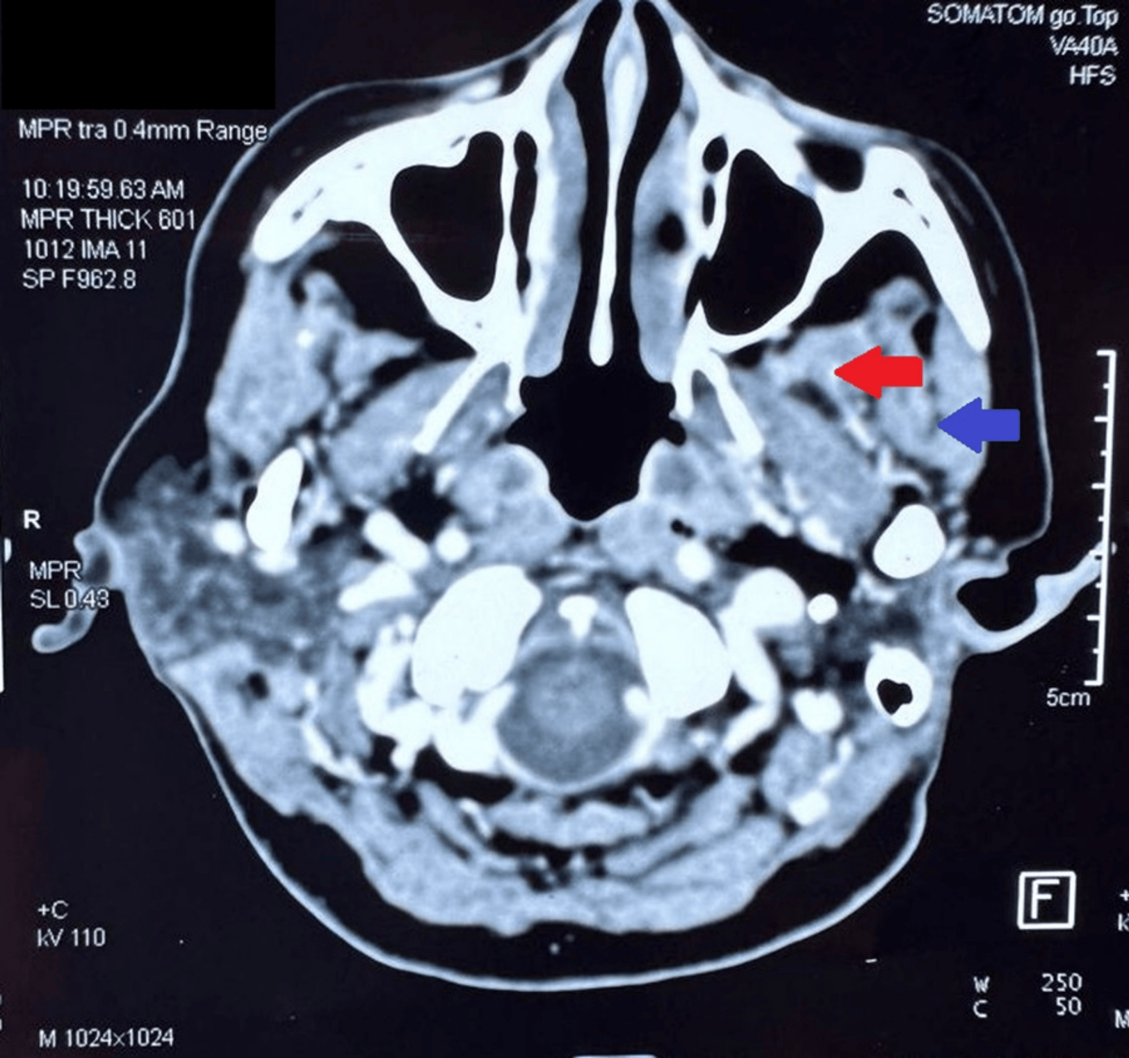
Oral cancer involving medial pterygoid and masseter in ITF post NACT Red arrow demonstrates the involvement of the medial pterygoid; Blue arrow demonstrates the involvement of the masseter. NACT: neoadjuvant chemotherapy; ITF: infratemporal fossa

**Figure 2:**
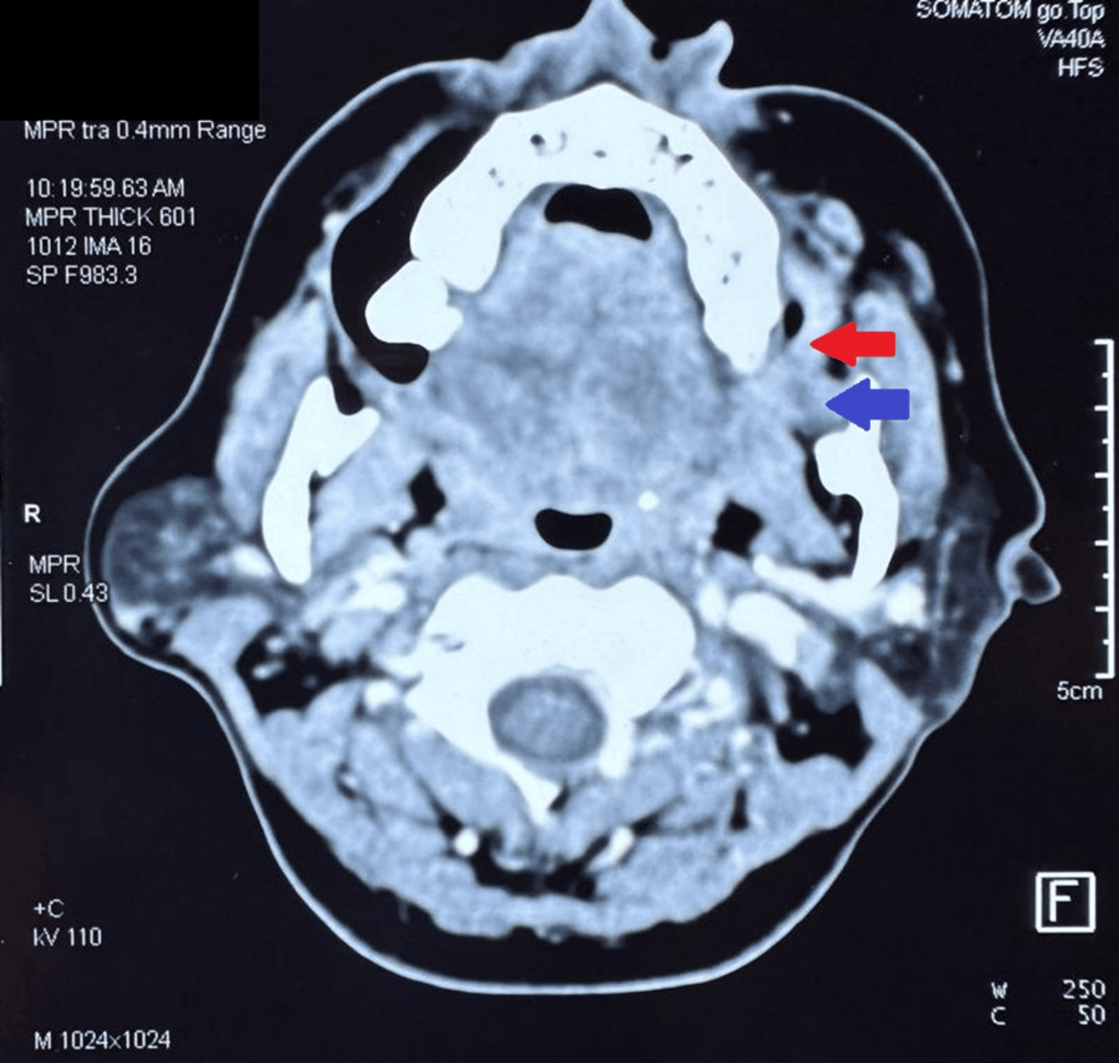
Tumor involving upper alveolus, retromolar trigone, and medial pterygoid in ITF post NACT Red arrow indicates the involvement of the upper alveolus; blue arrow indicates the involvement of the retromolar trigone and the medial pterygoid. NACT: Neoadjuvant Chemotherapy; ITF: infratemporal fossa

The clinical data of 96 patients with locally advanced oral cancers who received NACT from December 2018 to November 2022 were reviewed retrospectively and were classified into Stages T4a and T4b based on AJCC classification (2018). The outcomes following two cycles of NACT with cisplatin (100mg/m^2^) and paclitaxel (175mg/m^2^) administered on days 0, 21 were documented according to Response Evaluation Criteria in Solid Tumors (RECIST) version 1.1 criteria (Figures 1, 2). The outcomes were classified into partial response, stable disease, or progressive disease. Adverse events during chemotherapy were documented according to Common Terminology Criteria for Adverse Events (CTCAE) 5.0 [7].

All these patients underwent definitive surgery followed by adjuvant treatment in the form of either radiotherapy alone or radiotherapy plus chemotherapy. Surgery was performed by the same senior Head & Neck surgeon to minimize bias. In patients who responded to NACT, the access to surgical resection was easier, however, the extent of resection was the same as that marked before the administration of NACT. The resected specimens were subjected to histopathology examination to assess for tumor dimensions, the depth of invasion, tumor margins, neural or vascular invasion, bony erosion, and lymph node metastasis.

The compliance to adjuvant treatment, toxicity, and break in Adjuvant treatment if any was documented. A bimonthly clinical examination and contrast-enhanced computed tomography (CECT) of the head and neck was done at 2 months, 4 months, and 6 months post-completion of adjuvant treatment were done to assess the loco-regional control or distant metastasis and findings were documented.

Statistical analysis

Data was entered into a Microsoft Excel data sheet (Microsoft Corporation, Redmond, USA) and was analyzed using SPSS 22 version software (IBM Corp., Armonk, USA). Categorical data was represented in the form of frequencies and proportions. The Chi-square test was used as a test of significance for qualitative data. Continuous data was represented as mean and standard deviation.

Graphical representation of data: MS Excel and MS Word (Microsoft Corporation, Redmond, USA) were used to obtain various types of graphs. A P-value (probability that the result is true) of <0.05 was considered statistically significant after assuming all the rules of statistical tests.

Statistical software: MS Excel, SPSS version 22 (IBM SPSS Statistics, Somers NY, USA) was used to analyze data

## Results

Nonety-six patients were included in the study after fulfilling the inclusion criteria. 69 patients (71.9%) were females and 27 patients (28.1%) were males. The majority of the patients, 34 (32.3%), belonged to the age group of 40-50 years with a mean age of 49 with a standard deviation of 11. Among the oral cavity tumors, buccal mucosa tumors were predominant and accounted for 67 (69.8%) cases whereas tumors involving the alveolus were seen in 19 (19.8%) cases and 10 (10.4%) had retromolar trigone tumors. 68 patients (70.8%) were staged T4a and 28 patients (29.2%) were staged T4b based on the American Joint Committee on Cancer (AJCC) Staging 8th edition (Table 1).

**Table 1:** Distribution of subjects according to T4 stage

Stage	Frequency	Percent
T4a	68	70.8
T4b	28	29.2
Total	96	100.0

The tumors involved the left side predominantly in 68 (70.8 %) patients since this region is constantly exposed to tobacco quid which is sometimes used overnight. All the patients had clinically palpable lymph nodes. 80 patients (83.3%) were staged N1, 10 patients (10.2%) were staged N2a and 6 patients (6.3%) were staged N2b clinically.

Based on the NACT response, 29 patients (30.2%) had progressive disease, 32 patients (33.3%) had partial response, and the remaining 35 patients (36.5%) had stable disease (Table 2).

**Table 2:** Distribution of subjects according to NACT response NACT: Neoadjuvant Chemotherapy

NACT Response	Frequency	Percent
Progressive	29	30.2
Partial Response	32	33.3
Stable Disease	35	36.5
Total	96	100.0

NACT response was classified according to the stage. Among the 68 patients (70.8%) who belonged to stage T4a, 18 patients (26.5%) had progressive disease, 24 patients (35.3%) had a partial response and 26 patients (38.2%) had stable disease. Of the 28 patients (29.2%) staged T4b, 11 patients (39.3%) had progressive disease, eight patients (28.6%) had a partial response and nine patients (32.1%) had stable disease. On comparing the T stage with the NACT response, there was no statistically significant difference found between the T4a and T4b stages and response with a P-value of 0.461 (Table 3).

**Table 3:** Distribution of subjects according to T4 stage and response to NACT Statistical test used: Chi square test The Chi-square value for the provided table is 1.55, with a p-value of 0.461, which is not statistically significant. NACT: Neoadjuvant Chemotherapy

NACT Response	T4a		T4b		p-Value: 0.461
	N	%	N	%	
Progressive	18	26.5%	11	39.3%	
Partial Response	24	35.3%	8	28.6%	
Stable Disease	26	38.2%	9	32.1%	

Adverse events during chemotherapy were analyzed according to CTCAE 5.0. Grade 3 hematological toxicity was observed in 35 patients (36.5%). Febrile neutropenia occurred in 12 patients (12.5%) which required intensive care support. All the 12 patients had an uncomplicated recovery. Grade 3 non-hematological toxicity was observed in 25 patients (26%). Common grade 3 non-hematologic toxicities were diarrhea in 25 patients (24%), mucositis in 10 patients (10%), and hypokalaemia in 20 patients (19%). nine patients (9.4%) developed grade 1 skin rash during NACT. Liver enzyme derangement was noted in five patients (5.2%).

**Figure 3:**
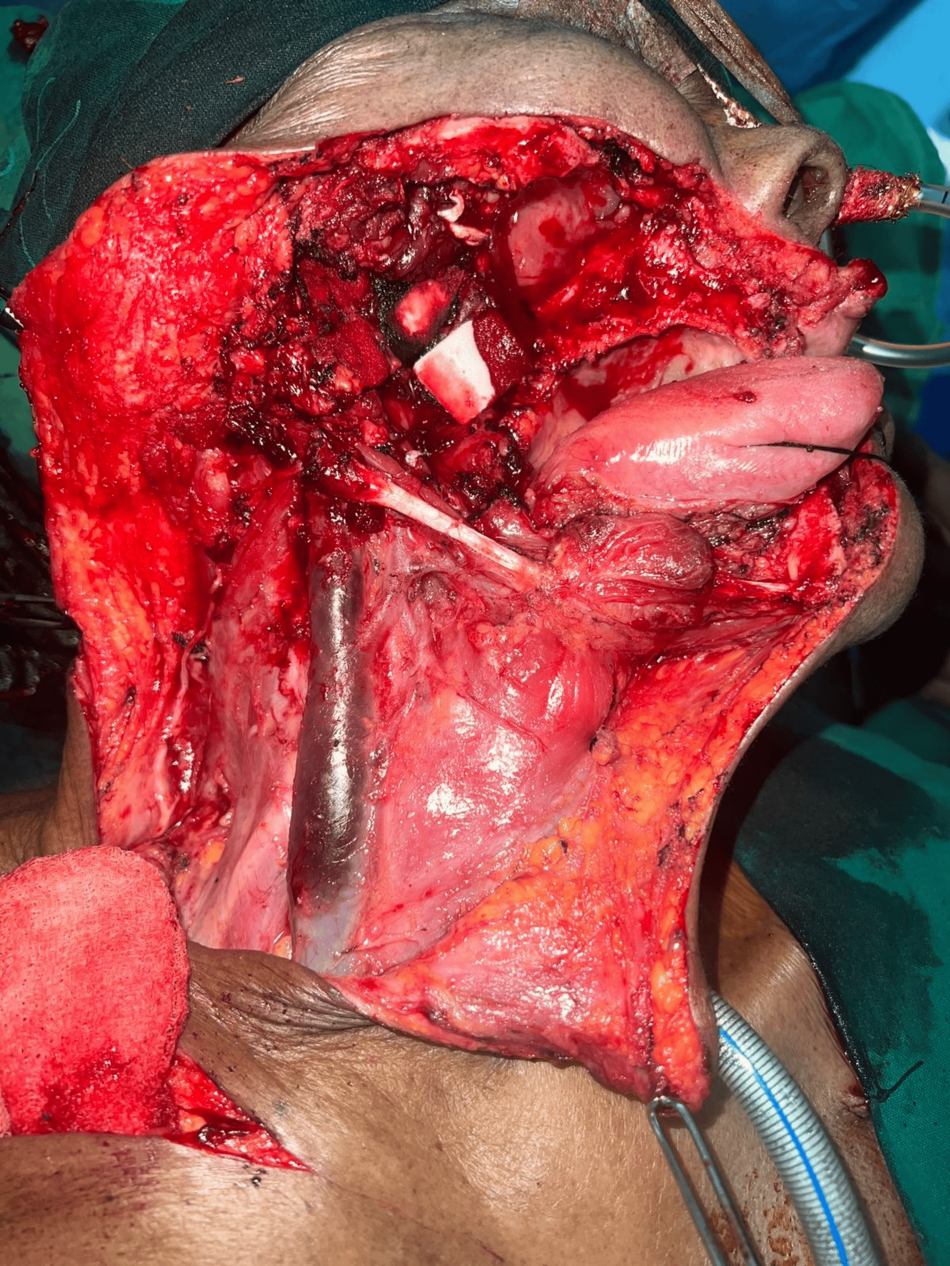
Right composite resection + infratemporal fossa clearance + modified radical neck dissection

**Figure 4:**
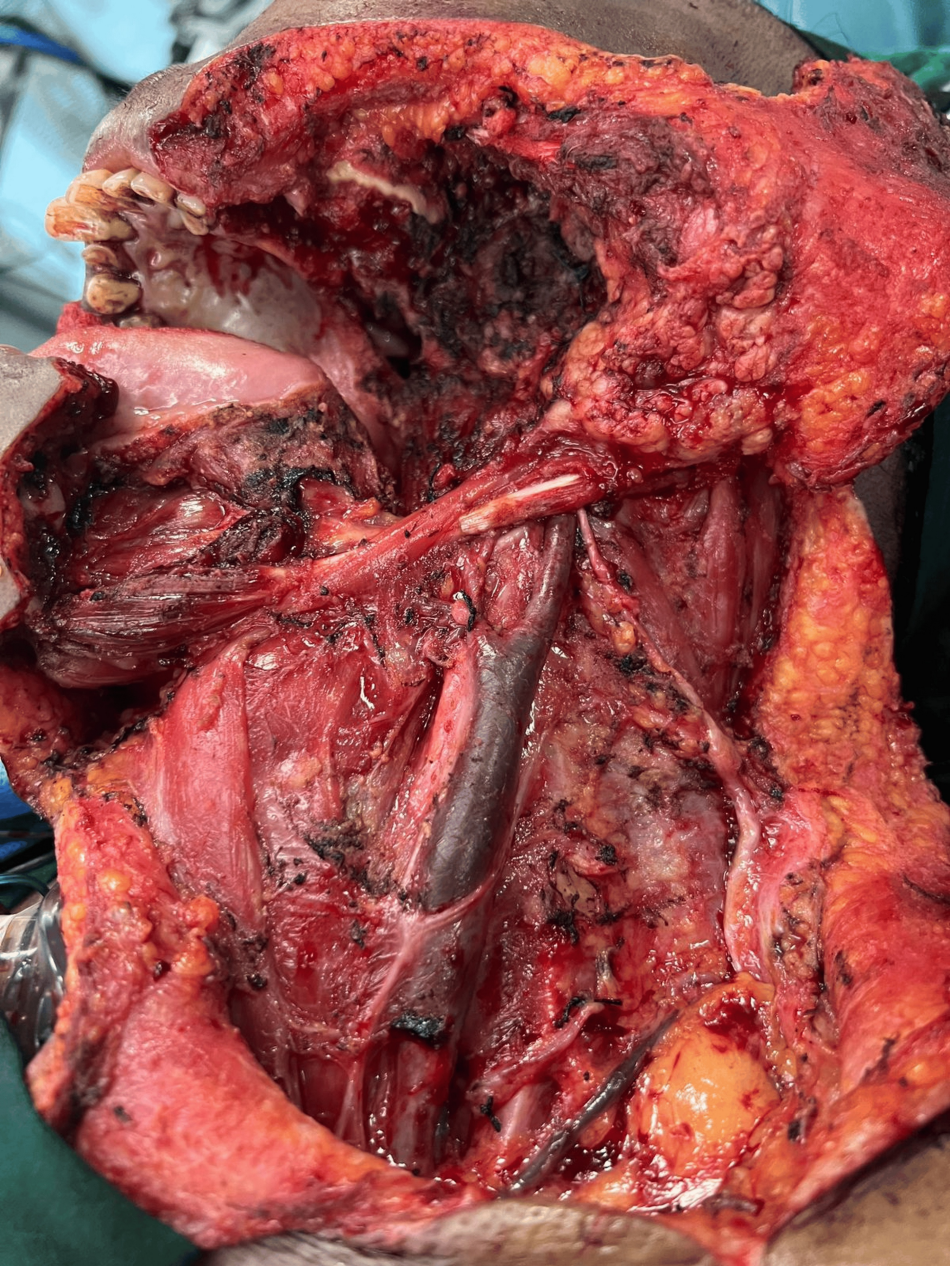
Left composite resection + infratemporal fossa clearance + modified radical neck dissection

All the patients underwent definitive surgery (composite resection + modified radical neck dissection + infratemporal fossa clearance + reconstruction) followed by adjuvant treatment in the form of either radiotherapy alone or radiotherapy plus chemotherapy (Figures 3, 4). All patients complied with adjuvant treatment and there was no break in adjuvant treatment in any of the patients.

The resected specimens were sent for histopathology examination. 36 patients (37.5%) had close margins with less than 5mm distance after formalin fixation on histopathology examination, of which 12 patients (33.3%) belonged to stage T4a and 24 patients (66.7%) belonged to stage T4b. 23 patients (24%) had positive margins, of which eight patients (34.8%) belonged to stage T4a and 15 patients (65.2%) belonged to stage T4b. Perineural invasion was seen in 10 patients (10.4%). Lymphovasular invasion was seen in nine (9.4%) patients. Histopathology examination of neck dissection specimens was done. 53 patients (55.2%) were staged N1, 18 patients (18.8%) were staged N2a, 14 patients (14.6%) were staged N2b while 11 patients (11.4%) had extranodal extension and were staged N3b.

With the data available for a minimum follow-up of 14 months, 57 patients (59.3%)were disease-free, 27 patients (28.1%) had a recurrence, four patients (4.2%) died due to disease, four patients (4.2%) died due to other causes, and four patients (4.2%) were lost to follow-up 6 months after completing treatment. Among the 27 patients who had a recurrence, 18 patients (66.7%) had local recurrence, eight patients (29.6%) had a locoregional recurrence, and one patient (3.7%) had distant metastasis. The recurrence was noted more in the T4b stage in 17 patients (60.7%) while 10 (16.2%) recurred in patients with T4a disease. A statistically significant difference was found between the T4a and T4b stages and recurrence with a P-value of 0.001 (Table 4).

**Table 4:** Distribution of subjects according to T4 stage and recurrence Statistical test used: Chi-square test. The chi-square value for the provided table is 16.20, with a p-value of 0.001, which is statistically significant.

Recurrence	T4a		T4b		p-value: 0.001
	N	%	N	%	
Absent	52	83.8%	11	39.3%	
Present	10	16.2%	17	60.7%	

Based on the NACT response, of the 29 patients who had progressive disease, 16 patients (55.2%) developed recurrence while seven patients (20%) of those with stable disease developed recurrence. Among the 32 patients with partial response to NACT, four patients (9.4%) developed recurrence. Among the 27 patients who had a recurrence, 15 patients (55.6%) had positive margins and 12 patients (44.4%) had close margins. Of the 11 patients who had extranodal extension, eight patients (72.7%) had locoregional recurrence within the 6 months of definitive treatment. Among the 10 patients who had perineural invasion, nine (90%) developed recurrence within the follow-up period. All patients with lymphovascular invasion on histopathology developed recurrence within 6 months of definitive treatment.

The factors predisposing to recurrence were close margins, positive margins, extranodal spread in lymph nodes, lymphovascular spread and perineural invasion.

## Discussion

The overall prevalence of OSCC has seen a downward trend in over the past few decades in most industrialized countries, but it remains a common malignancy in both males and females in south-central Asia and central and eastern Europe. Head and neck cancer accounts for nearly 30% of all cancers in India and almost 50% of head and neck cancers are oral cancer. The prevalence of oral cancer in our region is high [8]. 60-80% of patients in our region present with locally advanced disease - stage T3 and T4. The majority of the patients in this study were females belonging to the age group of 40-50 years. Oral cancer is becoming more common and hence it is mandatory to strengthen the diagnostic and therapeutic modalities [1]. The modality of treatment for patients with OSCC is composite resection with neck dissection and ITF clearance for advanced cases. Neck dissections are performed as elective procedures or when radiological or clinical evaluations reveal cervical nodal metastases. The stage of the tumor, the state of the resected margin, the depth of invasion, and the presence of cervical nodal metastases are important prognostic markers that impact these individuals.

A major challenge to head and neck surgeons across the globe is the management of locally advanced oral cancer staged T4 which carries a poor outcome. The T4b cancers were largely considered inoperable and offered palliative care only. Following a study by Liao et al. in 2007, few centers particularly in Asia have started venturing into selected T4b resections extending to infratemporal fossa and found good outcomes with infra sigmoid notch of mandible tumors and selected supranotch tumors with infratemporal fossa spread [9]. However, the challenge has been the absence of tumor barriers in the ITF, difficult access, and compact neurovascular contents making resection of extensive tumors difficult. Therefore NACT has been tried. The role of NACT has been controversial in these locally advanced tumors. It has been observed that resectable tumors do better with upfront surgery and those with difficult access or borderline inoperability tend to do better with NACT. However, a few patients fail to respond to chemotherapy making them inoperable thus making its role controversial. This study retrospectively analyzed the oncological outcomes of locally very advanced (T4a and T4b) OSCC treated by two cycles of NACT followed by definitive surgery and adjuvant treatment.

A few researchers have compared the results of upfront surgery with NACT followed by surgery in the literature. The majority of this research was conducted in Western countries, which also included individuals with locally advanced resectable cancer staged T3 and T4a [10]. On the contrary, our study only looked at T4 disease, and almost 30 % of the participants had ITF involvement and were staged T4b stage.

A few articles from Western countries have highlighted the preservation of the mandible by administering NACT in locally advanced oral cancers whereas loco-regional control (disease-free survival) and treatment-related complications were the primary endpoints of our study. In our study, radiotherapy and chemotherapy combined with radiotherapy were used as an adjuvant treatment after surgery. This was in contrast to research by Zhong et al. or Cohen et al., where the adjuvant treatment was limited to radiotherapy alone [11-12]. A large number of patients in our series had involvement of the ITF, where close margins of resection less than 5 mm after formalin fixation was present in 44.4% of cases, positive margins were prevalent on histopathology in 55.6% of cases and 11.4 % of patients had an extranodal extension. All these patients were administered adjuvant chemotherapy in addition to radiotherapy which is the standard practice in advanced oral cancers.

In our study, the NACT was administered as a three-week regimen of carboplatin and paclitaxel. 29 patients (30.2%) had progressive disease, 32 patients (33.3%) had partial response, and the remaining 35 patients (36.5%) had stable disease. None of the patients had a complete response. In contrast, 72% of patients had a partial response to NACT, while 8% had a complete response in a study done by Zhong et al [11]. This can be attributed to the three-drug regimen - docetaxel, cisplatin, and fluorouracil (TPF) - used in their study. The TAX323 & TAX324 trial also suggested a three-drug regimen that included cisplatin and docetaxel for NACT [12]. Studies by Cohen et al. and Licitra et al., however, did not demonstrate an improvement in the survival of patients receiving NACT [12, 13].

Studies by Zhong et al. and Bossi et al. included locally advanced resectable oral cancers (T3 & T4a), but our study included 30% of patients staged T4b and 70% of patients T4a [11, 14]. Another striking difference in our study is the administration of only two cycles for NACT whereas Cohen et al employed three cycles of NACT [11].

The disease-free survival rates observed in our study were 59% over a minimum follow-up period of 14 months. Zhong et al. had a disease-free survival of 62-63.6% in both NACT and upfront surgery in their study. Similar results have been documented by Bossi et al and studies in Italy, China, and India [14].

In our study, patients staged T4b had a higher frequency of locoregional recurrences (60%) in comparison to patients staged T4a (16%). A statistically significant difference (p-value: 0.001) in recurrence was found between the T4a and T4b stages in our study though all patients were subjected to two cycles of NACT. We also noted that 55.2% of the patients with progressive disease and 20% of the patients with stable disease developed recurrence while only 9.4% of the patients with partial response to NACT developed recurrence in the follow-up period. Similar observations were made by Patil et al., which revealed reasonably good locoregional control rates in advanced oral malignancies following the administration of NACT [6]. Research also demonstrates a 6-10% advantage when chemotherapy (ideally taxane-based) is administered to locally advanced oral malignancies in both adjuvant and neoadjuvant situations [14].

Factors predisposing to recurrence in our study were close margins, high involvement of ITF, extranodal spread, and perineural and lymphovascular invasion. Similar risk factors have been identified in other studies [13]. Lower rates of distant metastasis have been quoted in studies by Licitra et al. and Cohen et al. [12, 13], however, the sample size, power, and shorter follow-up duration did not facilitate similar observation in our study.

NACT provides a better surgical field in patients having locally advanced oral cancer due to a reduction in tumor volume and better access to surgical margins.

Limitations of the study

This was a single institutional study with a limited duration of follow-up (mean follow-up: 14 months). The other limitation of this study is that no randomization with stage-matched controls who did not receive NACT was done. A larger multi-institutional study with a control arm of stage and age-matched controls with randomized controls who do not receive NACT with a longer follow-up would be desirable.

## Conclusions

The responders to NACT have better locoregional control even in very advanced oral cancers (T4b) involving ITF. In resectable T4 tumors, upfront surgery is preferable as 30% of the patients do not respond to NACT. NACT induces a high response rate that may facilitate definitive surgery in locally advanced oral cancers. However, larger volume studies involving multiple institutions are required for a definitive protocol.
